# Psychedelics and Mental Health: A Population Study

**DOI:** 10.1371/journal.pone.0063972

**Published:** 2013-08-19

**Authors:** Teri S. Krebs, Pål-Ørjan Johansen

**Affiliations:** Department of Neuroscience, Faculty of Medicine, Norwegian University of Science and Technology (NTNU), Trondheim, Norway; Peking University, China

## Abstract

**Background:**

The classical serotonergic psychedelics LSD, psilocybin, mescaline are not known to cause brain damage and are regarded as non-addictive. Clinical studies do not suggest that psychedelics cause long-term mental health problems. Psychedelics have been used in the Americas for thousands of years. Over 30 million people currently living in the US have used LSD, psilocybin, or mescaline.

**Objective:**

To evaluate the association between the lifetime use of psychedelics and current mental health in the adult population.

**Method:**

Data drawn from years 2001 to 2004 of the National Survey on Drug Use and Health consisted of 130,152 respondents, randomly selected to be representative of the adult population in the United States. Standardized screening measures for past year mental health included serious psychological distress (K6 scale), mental health treatment (inpatient, outpatient, medication, needed but did not receive), symptoms of eight psychiatric disorders (panic disorder, major depressive episode, mania, social phobia, general anxiety disorder, agoraphobia, posttraumatic stress disorder, and non-affective psychosis), and seven specific symptoms of non-affective psychosis. We calculated weighted odds ratios by multivariate logistic regression controlling for a range of sociodemographic variables, use of illicit drugs, risk taking behavior, and exposure to traumatic events.

**Results:**

21,967 respondents (13.4% weighted) reported lifetime psychedelic use. There were no significant associations between lifetime use of any psychedelics, lifetime use of specific psychedelics (LSD, psilocybin, mescaline, peyote), or past year use of LSD and increased rate of any of the mental health outcomes. Rather, in several cases psychedelic use was associated with lower rate of mental health problems.

**Conclusion:**

We did not find use of psychedelics to be an independent risk factor for mental health problems.

## Introduction

Psychedelic plants have been used for celebratory, religious or healing purposes for thousands of years [1]–[3]. Use of psychedelics increased in the 1960s and has remained widespread in many parts of the world ever since. Over 30 million people living in the US have used lysergic acid diethylamide (LSD), psilocybin (magic mushrooms), and mescaline (peyote and other cacti) [4]. Common reasons for using psychedelics include mystical experiences, curiosity, and introspection [5]. The classical serotonergic psychedelics are not known to cause damage to the brain or other organs of the body, or cause withdrawal symptoms, elicit addiction or compulsive use [3], or cause birth defects or genetic damage [6]. Psychedelics often elicit deeply personally and spiritually meaningful experiences and sustained beneficial effects [7]–[12]. Psychedelics can often cause period of confusion and emotional turmoil during the immediate drug effects [13] and infrequently such adverse effects last for a few days after use. Psychedelics are not regarded to elicit violence [14] and dangerous behavior leading to suicide or accidental death under the influence of psychedelics is regarded as extremely rare [15]. LSD and psilocybin are consistently ranked in expert assessments as causing less harm to both individual users and society than alcohol, tobacco, and most other common recreational drugs [16]–[19]. Given that millions of doses of psychedelics have been consumed every year for over 40 years, well-documented case reports of long-term mental health problems following use of these substances are rare. Controlled studies have not suggested that use of psychedelics lead to long-term mental health problems [8], [9], [13], [20]. Here we evaluate the association between the use of psychedelics and mental health among US adults.

## Materials and Methods

### Ethics Statement

This study was exempt from review by our Regional Committee for Medical Research Ethics because all data are available in the public domain without any identification of personal information. The National Survey on Drug Use and Health (NSDUH) was approved by an institutional review board of the Research Triangle Institute.

### Source, Population and Data

The annual NSDUH survey provides estimates of substance use and mental health indicators from a randomly-selected sample representative of the general US civilian non-institutionalized adult population. The Substance Abuse and Mental Health Services Administration of the US Department of Health and Human Services is responsible for the NSDUH study design and methods of assessment. Trained interviewers met the randomly-selected participants in their homes, and participants listened to recorded questions via headphones and then entered their answers directly into a computer, providing a highly confidential and standardized setting. We pooled data from NSDUH survey years 2001 to 2004 because in these years participants were asked about symptoms of a range of psychiatric disorders and about whether they have been exposed to an extremely stressful event. We excluded half of the participants from year 2004 because of changes in the survey questions. We restricted the samples to adults aged 18 years and older because younger participants were asked different mental health questions than adults. The response rate was 78%. In addition, approximately 10% of participants were excluded from the public use data file, either because of excessive missing data on drug use or because they were excluded at random in order to increase anonymity. Detailed information on the sampling and data collection methods, including interview instructions and questionnaires, confidentiality and informed consent are available at the NSDUH website (http://oas.samhsa.gov/nsduh.htm).

### Use of Psychedelics

We counted participants as having any lifetime psychedelic use if they affirmed use of LSD, psilocybin, mescaline, or peyote. We also examined use of each of the substances separately. Mescaline and peyote was combined into one category “mescaline/peyote” because mescaline is the active substance in peyote cactus, but peyote was also examined separately. Information was also available on past year use of LSD, but not past year use of psilocybin or mescaline. LSD, psilocybin, and mescaline are all classical serotonergic psychedelics with main mechanism of action at the serotonin 2A receptor [3], [21].

### Mental Health Indicators

#### Serious psychological distress

The K6 scale provides a valid assessment of general psychological distress during the worst month of the past year, that are common to a broad range of psychiatric disorders, with strong accuracy in discriminating between people with and without one or more diagnoses from the Diagnostic and Statistical Manual of Mental Disorders, Fourth Edition (DSM-IV [22]) [23]. The K6 scale asks about frequency, using a 0 to 4 category scale, of six symptoms of psychological distress: feeling nervous, feeling hopeless, feeling restless or fidgety, feeling so sad or depressed that nothing could cheer you up, feeling everything was an effort, and feeling no good or worthless. A score of 13 or more on the K6 scale is the validated and recommended cut-point for serious psychological distress [23].

#### Mental health treatment

Past year mental health treatment was divided into four outcome variables: inpatient mental health treatment, outpatient mental health treatment, psychiatric medication prescription, and felt a need but did not receive mental health treatment. Inpatient mental health treatment included overnight stays for alcohol or drug problems at hospitals or rehabilitation centers. Outpatient mental health treatment included treatment for alcohol or drug problems at rehabilitation centers, mental health centers, emergency rooms, doctors’ offices, prisons or jails, or self-help groups. Data was not available on medication prescription for alcohol or drug problems. Needed but did not receive mental health treatment included respondents who felt that they needed treatment for alcohol or drug problems but did not receive any such treatment.

#### Psychiatric symptom indicators

Symptoms indicators for eight DSM-IV psychiatric disorders were evaluated using the short form of the World Health Organization Composite International Diagnostic Interview (CIDI-SF) [23]. The CIDI-SF consists of between three to eight questions per disorder and covers eight disorders: panic disorder, major depressive episode, mania, social phobia, general anxiety disorder, agoraphobia, posttraumatic stress disorder, and non-affective psychosis. We also examined each of the seven symptoms of non-affective psychosis individually (the cut-off for non-affective psychosis was two or more of the seven symptoms). The CIDI-SF appears to overestimate the rate of diagnoses, but most false-positive cases have some degree of the disorder even if they fail to meet full diagnostic criteria [23], [24]. We used the CIDI-SF to compare groups on symptom indicators, not to estimate prevalence of psychiatric diagnoses. We used standard scoring and cut-off points [25].

### Control Variables

We selected control variables based on associations with mental health in previous research [26]. Control variables consisted of a variety of sociodemographic, psychological, and drug use variables: age at interview (11 categories), gender, race/ethnicity (7 categories: non-Hispanic white, non-Hispanic black, non-Hispanic Native American, non-Hispanic Native Hawaiian or Pacific Islander, non-Hispanic Asian, non-Hispanic more than one race, Hispanic), education (4 categories: did not graduate high school; high school graduate; some college; college graduate), household income (4 categories: less than $20,000; $20,000 to $49,999; $50,000 to $74,999; $75,000 or more), marital status (2 categories: single; married), likes to test self with risky behavior (“How often do you like to test yourself by doing something a little risky?”; 4 categories: never, seldom, sometimes, always), lifetime exposure to an extremely stressful event (“Such as being in combat, being involved in a life-threatening accident, being involved in a disaster, being physically beaten or sexually abused, or any other event which was extremely upsetting or stressful”), and lifetime non-medical use of each of ten types of drugs: cannabis (marijuana), opiates (heroin, opiate pain relievers), cocaine, tranquilizers/sedatives (benzodiazepines, barbiturates), stimulants (amphetamine, methamphetamine, methylphenidate), MDMA (ecstasy), inhaled anesthetics (nitrous oxide, ether), alkyl nitrites (poppers), other inhalants (solvents, volatile chemicals), and PCP (phencyclidine). Additionally, in the analyses of past year use of LSD we also included as control variables past year use of the other drugs listed above, but with only one variable for any past year inhalant use because data on specific inhalants was not available.

### Data Analysis

We used multivariate logistic regression to calculate associations between the past year mental health indicators and use of psychedelics, including lifetime use of any psychedelics, lifetime use of LSD, psilocybin, mescaline/peyote, or peyote, and past year use of LSD. We also calculated the associations between the past year mental health indicators and lifetime use of any psychedelics in the presence or absence of other risk factors in stratified subgroups (sex, age, past year illicit drug use, lifetime exposure to an extremely stressful event). Participants with missing data on relevant mental health outcomes or past year illicit drug use were excluded.

The estimated associations between the use of psychedelics are presented as adjusted odds ratios (aOR), 95% confidence intervals (CI), and *p*-values. A statistically significant odds ratio greater than one indicates an association, and an odds ratio less than one indicates an inverse association. Because the mental health outcomes are all relatively uncommon, in this case, the odds ratio is a close approximation to the relative risk. For example, an adjusted odds ratio of 0.6 for a given outcome indicates that the rate of that outcome in psychedelic users is approximately 60% the rate in non-psychedelic users, after adjusting for control variables.

We used a standard alpha of 0.05; however any significant results should be considered in the context of the number of statistical analysis performed. It is typically recommended to have at least 10 events per predictor variable for multivariate logistic regression, although recent simulation studies suggest as few as 5 events per predictor variable is sufficient [27]. All the unstratified analyses had at least 10 events per predictor variable, with 21 to 379 events per predictor variable for mental health indicators besides the specific psychotic symptoms. In the stratified analyses of three of the more uncommon specific psychotic symptoms (“force inserting thoughts”, “force stealing thoughts”, “plot to harm you”) there were in some cases less than 10 and as few as 5 events per predictor variable. For all control variables the variance inflation factors were under 2.5, indicating little multi-collinearity. All calculations took into account the weighting variables and complex sample design variables of the NSDUH. For all calculations we used SPSS/PASW Statistics (version 18.0.3) with the Complex Samples Module.

## Results

### Characteristics of Psychedelic Users

The sample consisted of 130,152 respondents, of which 21,979 (13.4% weighted) reported lifetime use of any psychedelic. Tables 1 and 2 show the characteristics of the participants, according to lifetime use of any psychedelic. Compared to respondents with no lifetime use of any psychedelic, respondents with lifetime use of any psychedelic were more likely to be younger, male, white, Native American, or more than one race, have somewhat higher income and more education, not be married, like to test self by doing risky things, experienced an extremely stressful event, and to have used all classes of illicit drugs. For all the control variables, the differences between psychedelic users and non-users was statistically significant (Chi-square tests, for all *p*<0.001). Before adjusting for these confounding factors, psychedelic users had higher rates of all indicators of mental health problems.

**Table 1:** Characteristics of people who have used and not used psychedelics.

	Among used psychedelics		Among not used psychedelics		Used psychedelics, within each category
	wt %	N	wt %	N	wt %
**Total**	100%	21967	100%	108034	13.4%
**Age**					
18 to 25 years old	20.6%	11810	13.9%	51000	18.7%
26 years or older	79.4%	10157	86.1%	57034	12.5%
**Sex**					
Male	61.0%	12736	45.9%	48052	17.1%
Female	39.0%	9231	54.1%	59982	10.0%
**Race/ethnicity**					
White	85.7%	18399	69.5%	71732	16.0%
Hispanic	6.7%	1679	12.4%	14926	7.7%
Black	4.0%	621	12.2%	14331	4.8%
Asian	1.0%	250	4.3%	3843	3.4%
Native American	0.9%	399	0.4%	1060	25.2%
Native Hawaiian or Pacific Islander	0.2%	77	0.3%	492	9.6%
More than one	1.4%	542	0.9%	1650	20.0%
**Household income**					
Less than $20,000	17.2%	5403	20.2%	27777	12.2%
$20,000 to $49,000	36.3%	8616	38.0%	42340	13.2%
$50,000 to $74,999	19.6%	3664	18.0%	17953	15.0%
$75,000 or more	26.8%	4284	23.8%	19964	15.4%
**Education**					
Not high school graduate	13.2%	3727	18.0%	19174	10.2%
High school graduate	30.5%	7265	32.3%	36988	12.8%
Some college	29.7%	6757	24.3%	29775	15.9%
College graduate	26.6%	4217	25.4%	22056	14.0%
**Marital status**					
Not married	54.6%	14978	41.3%	62086	17.0%
Married	45.4%	6985	58.7%	45912	10.7%
**Likes to test self by doing risky things**					
Never	24.5%	4486	53.7%	48090	6.6%
Seldom	45.2%	9662	34.2%	40565	17.0%
Sometimes	27.3%	6780	11.1%	17174	27.6%
Always	3.1%	1017	1.1%	1922	31.1%
**Extremely stressful event**					
No	47.7%	10951	66.1%	71039	10.1%
Yes	52.3%	10938	33.9%	36361	19.3%
**Lifetime use of other drugs**					
Cannabis	98.2%	21542	33.0%	42705	31.5%
Opiates	46.2%	11249	7.7%	10958	48.1%
Cocaine	70.1%	14014	6.7%	6959	61.8%
Tranquilizers and sedatives	44.1%	8863	4.7%	5131	59.2%
Stimulants	41.8%	8648	3.7%	4455	63.3%
MDMA	24.7%	8074	1.2%	2680	75.9%
Inhaled anesthetics	28.5%	7515	1.6%	2735	74.0%
Alkyl nitrites	19.8%	3448	1.3%	1283	70.3%
Other inhalants	12.8%	2999	1.6%	2750	55.6%
PCP	21.0%	3752	0.4%	481	89.3%

Wt %, weighted percentage.

**Table 2:** Drug use in psychedelic users and non-users.

	Among used psychedelics		Among not used psychedelics		Used psychedelics, within each category
	wt %	N	wt %	N	wt %
**Total**	100%	21967	100%	108034	13.4%
**Psychedelics**					
LSD	80.1%	17486	0%	0	100%
Psilocybin	61.5%	14413	0%	0	100%
Mescaline/peyote	37.8%	6254	0%	0	100%
Peyote	19.6%	3120	0%	0	100%
LSD past year		1220		0	100%
**Lifetime use of other drugs**					
Cannabis	98.2%	21542	33.0%	42705	31.5%
Opiates	46.2%	11249	7.7%	10958	48.1%
Cocaine	70.1%	14014	6.7%	6959	61.8%
Tranquilizers and sedatives	44.1%	8863	4.7%	5131	59.2%
Stimulants	41.8%	8648	3.7%	4455	63.3%
MDMA	24.7%	8074	1.2%	2680	75.9%
Inhaled anesthetics	28.5%	7515	1.6%	2735	74.0%
Alkyl nitrites	19.8%	3448	1.3%	1283	70.3%
Other inhalants	12.8%	2999	1.6%	2750	55.6%
PCP	21.0%	3752	0.4%	481	89.3%

Wt %, weighted percentage.

### Logistic Regression Results

Tables 3 and 4 show the results of the multivariate logistic regression analyzes.

**Table 3:** Association between psychedelic use and mental health.

	Ever used psychedelics		Never used psychedelics			
	wt %	N	wt %	N	Adjusted OR^a^ (95% CI)	p
**Serious psychological distress in worst month of past year**						
K6-scale	15.5%	3826	7.5%	10389	1.0 (0.9–1.1)	0.72
**Mental health treatment in past year**						
Inpatient	2.9%	708	0.9%	1135	0.9 (0.7–1.2)	0.53
Outpatient	15.2%	3343	6.5%	7739	0.9 (0.8–1.0)	0.13
Medication	16.3%	3320	9.1%	9135	0.9 (0.8–1.0)	0.05
Needed but did not receive	11.9%	2979	4.2%	6320	0.9 (0.8–1.1)	0.31
**Symptoms of mental disorders in past year**						
Panic disorder	16.5%	4018	8.5%	10867	1.0 (0.9–1.1)	0.62
Major depressive episode	6.8%	1640	2.7%	3828	1.0 (0.8–1.2)	0.80
Mania	1.9%	407	0.7%	717	1.1 (0.8–1.6)	0.53
Social phobia	1.4%	302	0.6%	690	0.9 (0.7–1.3)	0.76
Generalized anxiety disorder	3.2%	739	1.4%	1770	0.9 (0.7–1.1)	0.31
Agoraphobia	1.4%	320	0.7%	853	1.0 (0.6–1.6)	0.90
Posttraumatic stress disorder	3.2%	649	1.2%	1456	1.0 (0.8–1.3)	0.86
Non-affective psychosis^b^	4.4%	658	1.8%	1451	0.8 (0.6–1.1)	0.21
**Specific psychotic symptoms in past year**						
Heard voices others could not	4.3%	639	2.1%	1628	1.0 (0.8–1.4)	0.82
Felt force taking over mind	2.3%	324	0.9%	736	**0.7 (0.5–1.0)**	**0.03**
Felt force inserting thoughts	1.0%	159	0.5%	328	0.7 (0.5–1.2)	0.23
Felt force steal thoughts	1.3%	230	0.7%	547	0.7 (0.5–1.2)	0.21
Force used special signals	3.3%	508	1.3%	1195	0.9 (0.7–1.2)	0.50
Believed plot to harm you	2.1%	332	0.9%	732	0.8 (0.5–1.2)	0.22
Saw vision others could not	4.0%	604	1.8%	1525	1.0 (0.7–1.3)	0.77

a Adjusted for age, gender, race/ethnicity, income, education, married, risky behavior, extremely stressful event, and ten types of lifetime drug use (cannabis/marijuana, opiates, cocaine, sedatives/tranquilizers, stimulants, MDMA/ecstasy, inhaled anesthetics, amyl nitrates, other inhalants, PCP).

b Data on symptoms of non-affective psychosis available only for years 2001–2002.

Bold indicates p<0.05.

**Table 4:** Association between use of LSD, psilocybin, mescaline, and peyote and mental health.

	Lifetime use								Past year use	
	LSD		Psilocybin		Mescaline/peyote		Peyote		LSD	
	Adjusted OR^a^(95% CI)	p	Adjusted OR^a^(95% CI)	p	Adjusted OR^a^(95% CI)	p	Adjusted OR^a^(95% CI)	p	Adjusted OR^b^(95% CI)	
**Serious psychological distress in worst month of past year**										
K6-scale	1.0 (0.9–1.1)	0.65	**0.8 (0.7–1.0)**	**0.009**	**0.9 (0.8–1.0)**	0.04	0.9 (0.7–1.0)	0.09	**0.7 (0.6–0.9)**	**0.01**
**Mental health treatment in past year**										
Inpatient	0.9 (0.6–1.2)	0.31	**0.8 (0.6–1.0)**	**0.04**	0.8 (0.6–1.0)	0.07	0.9 (0.6–1.2)	0.41	1.0 (0.6–1.5)	0.96
Outpatient	**0.9 (0.8–0.9)**	**0.002**	0.9 (0.8–1.0)	0.10	0.9 (0.8–1.1)	0.23	0.9 (0.7–1.1)	0.17	0.9 (0.7–1.2)	0.46
Medication	**0.9 (0.8–1.0)**	**0.04**	**0.8 (0.7–0.9)**	**<0.0001**	**0.8 (0.7–0.9)**	**0.004**	**0.8 (0.7–1.0)**	**0.01**	0.9 (0.7–1.2)	0.44
Needed but did not receive	1.0 (0.9–1.1)	0.47	0.9 (0.8–1.1)	0.44	**0.8 (0.7–0.9)**	**0.001**	0.8 (0.7–1.0)	0.08	1.0 (0.8–1.4)	0.93
**Symptoms of mental disorders in past year**										
Panic attacks	0.9 (0.9–1.1)	0.30	**0.9 (0.8–1.0)**	**0.006**	1.0 (0.9–1.2)	0.98	1.0 (0.8–1.1)	0.61	0.8 (0.6–1.0)	0.09
Major depressive episode	1.0 (0.8–1.1)	0.61	0.9 (0.8–1.1)	0.18	0.9 (0.7–1.0)	0.14	0.9 (0.7–1.2)	0.67	0.8 (0.5–1.1)	0.21
Mania	1.2 (0.9–1.8)	0.23	0.8 (0.6–1.0)	0.08	0.8 (0.6–1.2)	0.24	0.8 (0.5–1.2)	0.22	0.8 (0.5–1.3)	0.33
Social phobia	1.0 (0.6–1.5)	0.93	0.8 (0.5–1.1)	0.20	0.7 (0.5–1.0)	0.06	1.0 (0.6–1.6)	0.90	1.2 (0.6–2.4)	0.61
Generalized anxiety disorder	0.9 (0.7–1.1)	0.30	0.8 (0.7–1.1)	0.19	0.9 (0.7–1.1)	0.31	0.9 (0.7–1.3)	0.63	1.1 (0.6–1.7)	0.85
Agoraphobia	1.0 (0.6–1.7)	0.94	0.8 (0.5–1.2)	0.27	**0.6 (0.4–0.9)**	**0.005**	1.1 (0.7–1.7)	0.75	0.7 (0.3–1.4)	0.26
Posttraumatic stress disorder	1.2 (1.0–1.6)	0.08	0.9 (0.7–1.2)	0.41	0.9 (0.7–1.2)	0.36	0.9 (0.6–1.2)	0.41	1.0 (0.6–1.6)	0.92
Non–affective psychosis^c^	0.9 (0.7–1.2)	0.51	0.9 (0.6–1.2)	0.42	0.8 (0.6–1.1)	0.27	0.8 (0.6–1.1)	0.17	0.9 (0.6–1.3)	0.51
**Specific psychotic symptoms in past year**										
Heard voices	1.0 (0.7–1.3)	0.73	1.0 (0.8–1.3)	0.94	0.9 (0.6–1.3)	0.55	0.8 (0.6–1.3)	0.45	0.8 (0.6–1.3)	0.41
Felt force taking over mind	0.7 (0.5–1.0)	0.08	**0.6 (0.5–0.9)**	**0.004**	**0.7 (0.5–1.0)**	**0.04**	0.9 (0.5–1.4)	0.54	0.7 (0.4–1.3)	0.26
Felt force inserting thoughts	0.9 (0.6–1.5)	0.72	0.7 (0.4–1.2)	0.23	1.2 (0.7–1.8)	0.51	1.3 (0.8–2.3)	0.31	1.7 (0.8–3.5)	0.19
Felt force steal thoughts	0.9 (0.5–1.5)	0.62	0.8 (0.5–1.4)	0.48	0.8 (0.4–1.5)	0.47	0.7 (0.4–1.1)	0.08	1.3 (0.7–2.5)	0.36
Force used special signals	0.9 (0.7–1.3)	0.73	0.9 (0.7–1.3)	0.60	1.0 (0.8–1.4)	0.84	1.1 (0.8–1.5)	0.69	1.4 (1.0–1.9)	0.06
Believed plot to harm you	0.9 (0.6–1.3)	0.62	1.0 (0.6–1.5)	0.65	0.9 (0.6–1.5)	0.66	0.8 (0.4–1.4)	0.38	1.1 (0.6–1.9)	0.82
Saw vision	1.0 (0.8–1.3)	0.91	0.9 (0.7–1.3)	0.66	0.9 (0.7–1.2)	0.50	0.8 (0.5–1.0)	0.08	1.3 (0.8–2.1)	0.22

a Adjusted for age, gender, race/ethnicity, income, education, married, risky behavior, extremely stressful event, and ten types of lifetime drug use (cannabis/marijuana, opiates, cocaine, sedatives/tranquilizers, stimulants, MDMA/ecstasy, inhaled anesthetics, amyl nitrates, other inhalants, PCP).

b Adjusted for above variables plus nine types of past year drug use (cannabis/marijuana, opiates, cocaine, sedatives/tranquilizers, stimulants, MDMA/ecstasy, inhalants, PCP).

c Data on symptoms of non-affective psychosis available only for years 2001–2002.

Bold indicates p<0.05.

#### Serious psychological distress

Lifetime psychedelic use was not significantly associated with serious psychological distress in the worst month of the past year. Among the specific psychedelics, lifetime psilocybin use (aOR 0.8, *p* = 0.009), lifetime mescaline use (aOR 0.9, *p* = 0.04), and past year LSD use (aOR 0.7, *p* = 0.01) were associated with lower rates of serious psychological distress.

#### Mental health treatment

Lifetime psychedelic use was not significantly associated with any of the mental health treatment variables. Among the specific psychedelics there were a number of significant associations with lower rate of receiving or needing mental health treatment. Lifetime LSD use was significantly associated with a lower rate of outpatient mental health treatment (aOR 0.9, *p* = 0.002) and psychiatric medication prescription (aOR 0.9, *p* = 0.04). Lifetime psilocybin use was significantly associated with a lower rate of inpatient mental health treatment (aOR 0.8, *p* = 0.04) and psychiatric medication prescription (aOR 0.8, *p* = 0.00008). Lifetime mescaline/peyote use was significantly associated with a lower rate of psychiatric medication prescription (aOR 0.8, *p* = 0.004) and needed but did not receive mental health treatment (aOR 0.8, *p* = 0.001). Lifetime peyote use was significantly associated with a lower rate of psychiatric medication prescription (aOR 0.8, *p* = 0.01).

#### Psychiatric symptom indicators

Lifetime psychedelic use was not significantly associated with any of the eight past year psychiatric symptom indicators (aOR range 0.8 to 1.1), and lifetime psychedelic use was significantly associated with a lower rate of one of the seven psychotic symptoms (“Felt a force taking over your mind”: aOR 0.7, *p* = 0.03). Among the specific psychedelics, lifetime psilocybin use was significantly associated with a lower rate of symptoms of panic attacks (aOR 0.9, *p* = 0.006), and lifetime mescaline/peyote use was significantly associated with a lower rate of symptoms of agoraphobia (aOR 0.6, *p* = 0.005). Lifetime psilocybin use and lifetime mescaline/peyote use was significantly associated with a lower rate of one of the specific psychotic symptoms (“Felt a force taking over your mind”: psilocybin, aOR 0.6, *p* = 0.004; mescaline/peyote: aOR 0.7, *p* = 0.04).

#### Stratified samples

In a series of multivariate logistic regression analyzes stratified by gender (male; female), age (18 to 25 years; 26 and older), any past year illicit drug use (no; yes), or lifetime extremely stressful event ever (no; yes) there were no significant associations with lifetime psychedelic use and greater risk of any of the mental health outcomes. Rather, in twelve cases there was an association with psychedelic use and lower rate of various mental health outcomes; however, most of these cases had marginal statistical significance (0.05<*p*<0.01). Among females, psychedelic users had a lower rate of the psychotic symptom “felt force taking over mind” (aOR 0.5, 95% CI 0.3 to 0.7, *p* = 0.0005). Among younger people, psychedelic users had a lower rate of symptoms of generalized anxiety disorder (aOR 0.8, 95% CI 0.6 to 1.0, *p* = 0.03). Among older people, psychedelic users had a lower rate of psychiatric medications (aOR 0.9, 95% CI 0.8 to 1.0, *p* = 0.03) and the psychotic symptom “felt force taking over mind” (aOR 0.5, 95% CI 0.3 to 0.8, *p* = 0.01). Among people with past year illicit drug use, psychedelic users had a lower rate of inpatient mental health treatment (aOR 0.7, 95% CI 0.5 to 0.9, *p* = 0.02), needed mental health treatment (aOR 0.9, 95% CI 0.7 to 1.0, *p* = 0.04), symptoms of generalized anxiety disorder (aOR 0.7, 95% CI 0.5 to 1.0, *p* = 0.05), symptoms of agoraphobia (aOR 0.6, 95% CI 0.4 to 1.0, *p* = 0.05), and symptoms of posttraumatic stress disorder (aOR 0.7, 95% CI 0.5 to 1.0, *p* = 0.02). Among people without a lifetime extremely stressful event, psychedelic users had a lower rate of symptoms of psychosis (aOR 0.5, 95% CI 0.3 to 0.9, *p* = 0.03) and the psychotic symptoms “felt force inserting thoughts” (aOR 0.4, 95% CI 0.2 to 0.9, *p* = 0.02) and “felt force steal thoughts” (aOR 0.3, 95% CI 0.1 to 0.7, *p* = 0.008).

#### Native americans

Native Americans reported a high rate of lifetime psychedelic use (25%, weighted), with a high rate of lifetime peyote use (14%, weighted). However, less than 1% of lifetime psychedelic users and less than 3% of lifetime peyote users were Native Americans. Many Native Americans use peyote within legally-protected religious practice [24]. Excluding Native Americans changed the adjusted odds ratios on average less than 2%, and mescaline/peyote use was no longer statistically significantly associated with lower rate of the specific psychotic symptom “felt a force taking over your mind” (aOR = 0.7, *p* = 0.06).

#### Missing data

On each analysis, less than 2% of participants were missing data. Including participants with missing data by setting missing data to “no” or “0” had a minimal effect on the results (less than 4% change in adjusted odds ratios, on average) and had no effect on statistical significance.

## Discussion

### Lack of Associations with Mental Health Problems

We found no relation between lifetime use of psychedelics and any undesirable past year mental health outcomes, including serious psychological distress, mental health treatment (inpatient, outpatient, medication, felt a need but did not receive), or symptoms of panic disorder, major depressive episode, mania, social phobia, generalized anxiety disorder, agoraphobia, posttraumatic stress disorder, or non-affective psychosis. In addition to not being significantly different from no association, in all cases the calculated adjusted odds ratios (aOR) were small (for all, psychedelic use aOR ≤1.2). Stratifying by age, gender, past year illicit drug use, or lifetime extremely stressful event did not substantially change the results of any of the logistic regression analyses. Likewise, lifetime use of LSD, psilocybin, mescaline, or peyote, or past year use of LSD, was not associated with a higher rate of mental health problems. There were a number of weak associations between use of any psychedelic or use of specific psychedelics and lower rate of mental health problems; these results might reflect beneficial effects of psychedelic use, relatively better initial mental health among people who use psychedelics, or chance “false positive” findings. Our results are consistent with assessments of the harm potential of psychedelics [28], [29] and with information provided by UN, EU, US, and UK official drug education programs [15], [30]–[34], insofar as these sources do not conclude that psychedelics are demonstrated to cause lasting anxiety, depression, or psychosis.

### Limitations

This study had a retrospective, cross-sectional design, making it impossible to draw causal inferences. Many potentially important risk factors, such as family mental health history, were not available. Longitudinal data were not available on mental health or other factors before psychedelic use. We cannot exclude the possibility that use of psychedelics might have a negative effect on mental health for some individuals or groups, perhaps counterbalanced at a population level by a positive effect on mental health in others. We did not adjust for multiple comparisons, so some of the associations with weak statistical significance are likely due to chance. Screening questions, rather than diagnostic interviews, were used as symptom indicators. Self-reports of drug use behaviors and mental health questions could be influenced by memory errors and under-reporting; however, a 14-year longitudinal study reported good consistency over time in reporting of LSD use [35]. Dosage and purity of street drugs is often unknown, and in particular substances sold as mescaline often contain LSD or other substances [36]. A small group (<2%) of US adults in prison, hospital, or military service were not included in the NSDUH sampling. We did not examine active drug or short-term effects.

### Clinical Studies in Healthy Volunteers

The lack of association between the use of psychedelics and indicators of mental health problems in this large population survey is consistent with clinical studies in which LSD or other psychedelics have been administered to healthy volunteers [13]. Eight recent double-blind, placebo-controlled studies of psilocybin in healthy volunteers, with follow-up between 8 and 16 months, reported “no subsequent drug abuse, persisting perception disorders, prolonged psychosis or other long term impairment of functioning” [20]. And two other recent clinical trials of psilocybin in 54 healthy volunteers found no evidence of lasting adverse effects [8], [9].

### Cross-sectional and Case-control Studies

A case-control study of Native Americans failed to find any evidence of cognitive or mental health deficits among people who regularly used peyote in religious services compared to those who did not use peyote, rather total lifetime peyote use (mean 300 occasions, range 150–500) was associated with overall better mental health [37]. Likewise, a longitudinal case-control study found that people who had each used the shamanic beverage ayahuasca, containing the psychedelic dimethyltryptamine (DMT) which is chemically similar to psilocybin, in over 360 religious ceremonies scored significantly lower on all psychopathology measures compared to people who regularly participated in non-psychedelic religious groups, both at baseline and at one year follow-up [38]. A population study reported that any lifetime use of cocaine and/or psychedelics was associated with prior lifetime history of two or more of 15 psychotic symptoms, but not one psychotic symptom [39]; in this study cocaine and amphetamine use were not included as control variables in the analyzes of psychedelic use, although both cocaine and amphetamine use were reported to be associated with psychotic symptoms; although the data came from a 10-year longitudinal study, data were aggregated over all time periods and no distinction was made between psychotic symptoms occurring before or after onset of psychedelic use. Another retrospective population study did not find an association between any lifetime “psychedelic” use and panic attacks or depression, but did report an association between dependence on “psychedelics” and panic attacks [40]; however, in this study the number of events was small, and the dissociative anesthetic PCP was included as a psychedelic, even though it is well known that PCP has quite different subjective effects, dependence potential, and neurobiological mechanisms than the serotonergic psychedelics. A follow-up of 29 patients with first-break psychosis attributed to LSD use found that these individuals were “essentially similar” to first-break psychosis patients with no LSD use in terms of premorbid adjustment, course of illness, and family history of inpatient treatment, and course of illness [41] (see also [28]).

### “Flashbacks” and Perceptual Phenomena

In this study, lifetime use of psychedelics and past year use of LSD was not associated with past year symptoms of visual phenomena (“seeing something others could not”), panic attacks, psychosis, or overall serious psychological distress. Thus, our findings does not support either the idea of “flashbacks” described in extreme cases as recurrent psychotic episodes, hallucinations, or panic attacks, or the more recent “hallucinogen persisting perceptual disorder” (HPPD) described as persistent visual phenomena with accompanying anxiety and distress. All of the purported symptoms of HPPD are also present in people who have never used psychedelics [42], [43]. Occasional visual phenomena are common in the general population [44], [45], especially among people with anxiety disorders [46]. Recent randomized controlled trials with psilocybin do not report any cases of “flashbacks” or persistent visual phenomena [8], [9], [20]. Interviews with over 500 regular participants in Native American peyote ceremonies did not identify anyone with “flashbacks” or persistent visual symptoms [37]. Interviews with 120 adults in the US complaining of persistent visual symptoms found that only 5% had ever used LSD (in comparison, over 10% of the general US adult population has used LSD [4]) and there did not seem to be any relationship between drug use and visual symptoms [47]. Only two small studies have reported higher rates of visual symptoms in LSD users compared to non-users [42], [43]. Both studies had serious methodological problems: participants in both studies were psychiatric inpatients who knew that the purpose of the studies was to document harms from LSD; the LSD group and the control groups were not matched on other drug use or psychiatric diagnosis; and in the first study several of the people included in the LSD group were later found to have epilepsy, panic, anxiety, affective disorder, or temporoparietal abnormalities that may be related to visual symptoms [48]. In case reports of “flashbacks” or HPPD, symptom onset is often weeks, even years, after last psychedelic use, and a causal relationship between persistent perceptual symptoms and use of psychedelics remains unproven. Overall, the validity of the HPPD diagnosis remains scant. HPPD appears to fit within the somatic symptom disorders [49]. In an illustrative case example, a young man was diagnosed with HPPD by the originator of the diagnosis; symptoms began in conjunction with major life changes and several weeks after taking LSD; on initial consultation with physicians, he was told that his vision was fine and somatization disorder was implied; he improved after psychotherapy for his depression and worries, and reassurance that his visual experiences were ordinary perceptual phenomena that most people ignore [50].

### Comments on Case Reports

Case reports of long-term psychiatric problems attributed to LSD, include psychosis, panic attacks, other anxiety disorders, and depression [3], [51]. There are very few case reports of prolonged psychiatric symptoms following psilocybin or mescaline [13], [52]. Almost all claims of psychiatric harm caused by peyote have been found on examination of medical records to be due to pre-existing schizophrenia or other causes [53], [54]. Several issues are important to keep in mind when considering case reports [13], [51]. 1) Adverse effects of psychedelics are usually short-lived; serious psychiatric symptoms following psychedelic are typically resolved within 24 hours or at least within a few days. 2) Both mental illness and psychedelic use are prevalent in the population, likely leading to many chance associations; for instance, about 3% of the general public will have a psychotic disorder sometime in their lives [55]. 3) The typical onset period of both mental illness and psychedelic use occurs in late adolescence and early adulthood, again possibly leading to mistaken causal inferences. 4) Most case reports do not rule-out preexisting psychiatric difficulties, life stresses, or use of other drugs. Many psychiatric disorders are believed to be heavily influenced by genetics and earlier experiences, even if symptoms are often first triggered by a stressful event. Note, however, that people with first-episode psychosis often have no apparent family or personal history of mental illness, whether or not if they have previously used psychedelics [41]. 5) Because of the striking subjective effects of psychedelics, some people attribute psychiatric symptoms to the use of psychedelics even if the symptoms started months or years later. 6) Some health professionals may have a biased view since they meet people with mental health problems and have little or no contact with the majority of psychedelic users. 7) Caution should be used when generalizing from LSD to other psychedelics because of emerging evidence of unique effects of LSD [56]. 8) Case reports of mental health problems following psychedelics are often comparable to case reports of mental health problems linked to intensive meditation [57]–[61], visiting holy sites [62], [63], or viewing beautiful artwork and sublime natural scenes [64].
